# Effect of Butyric Acid in the Proliferation and Migration of Junctional Epithelium in the Progression of Periodontitis: An In Vitro Study

**DOI:** 10.3390/dj9040044

**Published:** 2021-04-16

**Authors:** Taichi Ishikawa, Daisuke Sasaki, Ryo Aizawa, Yu Shimoyama, Matsuo Yamamoto, Tarou Irié, Minoru Sasaki

**Affiliations:** 1 Division of Molecular Microbiology, Department of Microbiology, Iwate Medical University, 1-1-1 Idai-dori, Yahaba-Cho, Shiwa-Gun, Iwate 028-3694, Japan; yushimo@iwate-med.ac.jp (Y.S.); msasaki@iwate-med.ac.jp (M.S.); 2 Division of Periodontology, Department of Conservative Dentistry, School of Dentistry, Iwate Medical University, 1-3-27 Chuo-dori, Morioka, Iwate 020-8505, Japan; daisukes@iwate-med.ac.jp; 3 Department of Periodontology, School of Dentistry, Showa University, 2-1-1 Kitasenzoku, Ohta-ku, Tokyo 145-8515, Japan; r-aizawa@dent.showa-u.ac.jp (R.A.); yamamoto-m@dent.showa-u.ac.jp (M.Y.); 4 Division of Anatomical and Cellular Pathology, Department of Pathology, Iwate Medical University, 1-1-1 Idai-dori, Yahaba-Cho, Shiwa-Gun, Iwate 028-3694, Japan; tarou@iwate-med.ac.jp

**Keywords:** adhesion molecules, butyric acid, junctional epithelium, periodontopathic bacteria

## Abstract

Purpose: To elucidate the effects of butyric acid (BA), a metabolite of bacteria involved in periodontitis, and a possible enhancer of the junctional epithelial cells. Methods: A murine junctional epithelial cell line, JE-1, was used to assess the effects of sodium butyrate (NaB) as BA. Cell proliferation, migration and attachment were analyzed. Additionally, gene and promoter expression analysis was performed, i.e., cap analysis of gene expression (CAGE) and gene ontology (GO) term enrichment analysis. Results: NaB affected junctional epithelial cell proliferation, migration and attachment. A high concentration of NaB caused cell death and a low concentration tended to promote migration and adhesion. CAGE analysis revealed 75 upregulated and 96 downregulated genes in the cells after 0.2 mM NaB stimulation for 3 h. Regarding GO term enrichment, the genes upregulated >4-fold participated predominantly in cell migration and proliferation. The results of this study suggest that BA produced from periodontopathic bacteria is involved in periodontal tissue destruction at high concentrations. Furthermore, at low concentrations, BA potentially participates in periodontal disease progression by increasing proliferation, migration and attachment of the junctional epithelium and thereby increasing epithelial down-growth.

## 1. Introduction

Periodontitis is a chronic multifactorial inflammatory disease associated with dysbiotic dental plaque biofilms [1]. The disease has been associated with systemic diseases such as diabetes mellitus [2,3,4], cardiovascular disease [5,6,7], rheumatoid arthritis [8,9], and neurological disorders such as Alzheimer’s disease [10,11,12]. Furthermore, previous studies have associated periodontal diseases with the risk of various human malignant neoplasms, such as poorly differentiated oral squamous cell carcinoma [13,14], as well as preterm birth and low birth weight [15,16]. The onset and progression of periodontal disease generally leads to periodontitis via gingivitis due to the formation of dental plaque on periodontal tissue. As the lesion progresses, the periodontal ligament is destroyed by inflammation and the junctional epithelium enters a state of attachment loss in which the epithelium progresses and propagates onto the cementum towards the root apex side. Thereafter, attachment loss continues from the crown side, and a periodontal pocket is formed between the tooth surface and the gingiva [17,18]. The majority of previous studies have focused on pathogenic factors, such as fimbriae, lipopolysaccharides and exotoxins, of periodontopathic bacteria represented by the “red complex” species (namely *Porphyromonas gingivalis*, *Treponema denticola* and *Tannerella forsythensis*). In contrast, few studies have investigated the metabolites of periodontopathic bacteria.

Previous studies have reported the presence of 0–14 mM butyric acid (BA) in the subgingival plaque [19,20]; large amounts of this carboxylic acid are produced by periodontopathic bacteria such as *P. gingivalis* and *Fusobacterium nucleatum* [21]. *P. gingivalis* is one of the “red complex” species and *F. nuclatum* is one of the “orange complex” species. Both have a high and moderate risk of periodontitis, respectively. BA has been reported to induce apoptosis in human, murine B and murine T lymphocytes within periodontal tissue [22,23]. Additionally, the results of our previous studies indicate that BA enhances the migration of ameloblastoma via laminin 332 [24,25]. On the other hand, being a short-chain fatty acid, BA is beneficial as a major energy source for intestinal epithelial cells. Some intestinal bacteria metabolites may reach the rest of the body through the intestinal tissues and bloodstream; they may then affect the immune response of a host through the control of regulatory T cells (Tregs) [26]. Indeed, BA derived from Clostridium species, which are part of the human intestinal flora, induces the differentiation of Tregs in the intestinal tract [27]. Tregs play a major role in negatively controlling the immune response of autoreactive T cells; they are important in avoiding damage due to excessive autoimmune responses. In a mouse model of colitis, BA-induced Tregs have been shown to suppress colitis pathogenesis [28]. Thus, BA is considered to be beneficial in the intestinal tract but harmful in the oral cavity.

Given the potentially harmful effects of BA on oral health, it is necessary to elucidate the effects of BA on periodontal tissue. Some studies have suggested that BA is involved in periodontal disease [29,30]. BA stimulation was found to reduce the expression of cell adhesion factors, namely, integrin α6 and integrin β4, but increase intercellular adhesion molecule-1 (ICAM-1) in the gingival epithelium and cause tissue destruction [30]. In research conducted to date, gingival epithelial cell lines have been studied rather than the junctional epithelium because the former cell lines, such as Ca9-22, are considered to be an established model for the study of periodontal disease [31,32,33]. In these studies, which tested the cell adhesion ability of periodontopathic bacteria, the type of *P. gingivalis* and its fimbriae as well as the S-layer protein of *T. forsythensis* were important in the early stage of oral infections including periodontal disease. Importantly, however, the gingival and junctional epithelium differ in their development processes and they are thought to differ in cell responsiveness [34,35]. Recently, a junctional epithelial cell line has been established; hence, it has become possible to use the cell line in research and to study its differences from gingival epithelial cell lines [36].

Currently, there remains a lack of research regarding the effects of BA on the junctional epithelium. Therefore, in the present study, we investigated these effects by examining the cell proliferation, migration, attachment and gene transcription of junctional epithelial cells exposed to BA.

## 2. Materials and Methods

### 2.1. Cell Culture

The junctional epithelial cell line JE-1, previously established by Matsuo Yamamoto in Showa University, Tokyo, Japan [36], was cultured in CnT-Prime (CELLnTEC, Bern, Switzerland) supplemented with 1% penicillin–streptomycin mixed solution (Nacalai Tesque, Kyoto, Japan). The cell line was incubated in a humidified atmosphere with 5% CO_2_ at 37 °C. Unless otherwise stated, cells from 80% confluent cultures were used following detachment with TrypLE Express (Gibco, MA, USA).

### 2.2. Cell Proliferation and Inhibition Assay

A cell proliferation and inhibition assay was performed using a Cell Counting Kit-8 (Wako, Osaka, Japan) following the manufacturer’s instructions. The cells were seeded onto 96-well plates (Corning, NY, USA) at 1 × 10^3^ cells/well in 100 µL of CnT-Prime. Cells were pre-incubated for 1 day before the assays began. After pre-incubation, the medium was changed to fresh CnT-Prime with various concentrations of NaB (0.02–20 mM; Wako) or fresh medium only (as a control), and then the cells were incubated for 7 days at 37 °C in 5% CO_2_. The estimated number of cells was recorded every 24 h. A SpectraMax M2 (Molecular Devices) was used to measure the optical density of the plate at a wavelength of 450 nm with a reference wavelength of 600 nm.

### 2.3. Migration Assay

A migration assay was conducted following a previously published method [37,38]. Briefly, 1 × 10^5^ cells/well were plated in 24-well plates with 500 µL of CnT-Prime and then incubated for 2 days at 37 °C in 5% CO_2_. Subsequently, the monolayer was scratched with a pipette tip and washed with phosphate-buffered saline (PBS) to remove non-adherent cells. The cells were then cultured in CnT-Prime supplemented with 0.02–20 mM NaB or fresh medium only (as a control). Finally, the cells were photographed at 0 h and 24 h by using fluorescence microscopy BZ-9000 (Keyence, Osaka, Japan).

### 2.4. Cell Attachment Assay

A cell attachment assay was performed according to a previously reported method [39,40]. Briefly, cells were seeded onto 96-well culture plates (Maxisorp, Nunc, Roskilde, Denmark) at 1 × 105 cells/well in a culture medium with various concentrations of NaB (0.02–20 mM) or fresh medium only (as a control) and then incubated for 60 min at 37 °C in 5% CO_2_. Thereafter, the wells were washed three times with PBS and attached cells were fixed with 4% paraformaldehyde phosphate buffer solution (Wako) for 15 min. They were then stained overnight with 0.5% toluidine blue O (Wako). Subsequently, the plates were washed three times with PBS and then 100 µL of 2% SDS was added to each well to release the blue dye. A SpectraMax M2 (Molecular Devices) was used to measure the optical density at a wavelength of 620 nm.

### 2.5. Real-Time Quantitative Reverse-Transcription Polymerase Chain Reaction

Cells were disseminated onto 24-well plates at 1.0 × 10^5^ cells/well in 500 µL of CnT-Prime for 24 h. The medium was then changed to either fresh medium containing various concentrations of NaB or fresh medium only (as a control), and the cells were incubated for 6 h at 37 °C in 5% CO_2_. After this treatment, total RNA was extracted from the cells using an RNeasy Mini Kit (Qiagen, Copenhagen, Denmark) in accordance with the supplied manufacturer’s instructions. The cDNA was synthesized with PrimeScript RT Master Mix (TaKaRa Bio., Shiga, Japan), and a real-time quantitative reverse-transcription polymerase chain reaction (qPCR) assay was performed using a Thermal Cycler Dice Real Time System (TaKaRa) following the manufacturer’s instructions. TB Green Premix Ex Taq II (TaKaRa) was used for the qPCR reaction. Primer sets, which were based on sequences for ICAM-1, integrin α6, integrin β4 and glyceraldehyde-3-phosphate dehydrogenase (GAPDH), were purchased from TaKaRa. The primers for Gapdh were used as an endogenous control. The cDNA amplification conditions were as follows: 95 °C for 30 s, followed by 40 cycles at 95 °C for 5 s and 60 °C for 30 s. Dissociation was performed to confirm the specificity of primers.

### 2.6. Gene and Promoter Expression Analysis

Gene and promoter expression analysis was performed according to previously published methods [41]. Cap analysis of gene expression (CAGE) library preparation, sequencing, mapping and gene expression were conducted on each RNA sample (DNAFORM). In brief, RNA quality was assessed with a Bioanalyzer (Agilent, CA, USA) to ensure that the RNA integrity number was >7.0 and the A260/280 and 260/230 ratios were >1.7. First-strand cDNAs were transcribed to the 5′-ends of capped RNAs and attached to CAGE “bar code” tags; the sequenced CAGE tags were mapped to the mouse vM24 genome using BWA software (v0.5.9) after discarding ribosomal or non-A/C/G/T base-containing RNAs. The CAGE-tag 5′ coordinates were inputted for CAGEr clustering [42] using the Paraclu algorithm [43] with the default parameters. The upregulated genes in 0.2 mM NaB-stimulated cells relative to unstimulated cells were identified with a threshold log2 fold change >2.0.

### 2.7. Gene Ontology (GO) Term Enrichment Analysis of CAGE Data

The differences in gene expression between unstimulated cells and those stimulated with 0.2 mM NaB for 3 h were detected using the DESeq2 package (v1.20.0). GO term enrichment analysis was performed using the DAVID database (https://david.ncifcrf.gov/, 2 November 2020).

### 2.8. Statistical Analysis

Results are presented as means and standard deviations. Significant differences were determined using one-way ANOVA followed by Tukey’s post hoc test. For the cell proliferation and inhibition assay, migration assay, cell attachment assay and qPCR, *p* < 0.05 was considered to be statistically significant. For GO term enrichment analysis, the p value is the threshold of the EASE score, a modified Fisher exact p value, for gene enrichment analysis. Following the DAVID database, *p* < 0.1 was considered to be statistically significant in GO analysis.

## 3. Results

### 3.1. Butyric Acid Has Concentration-Dependent Positive and Negative Effects on Cell Proliferation

**Figure 1:**
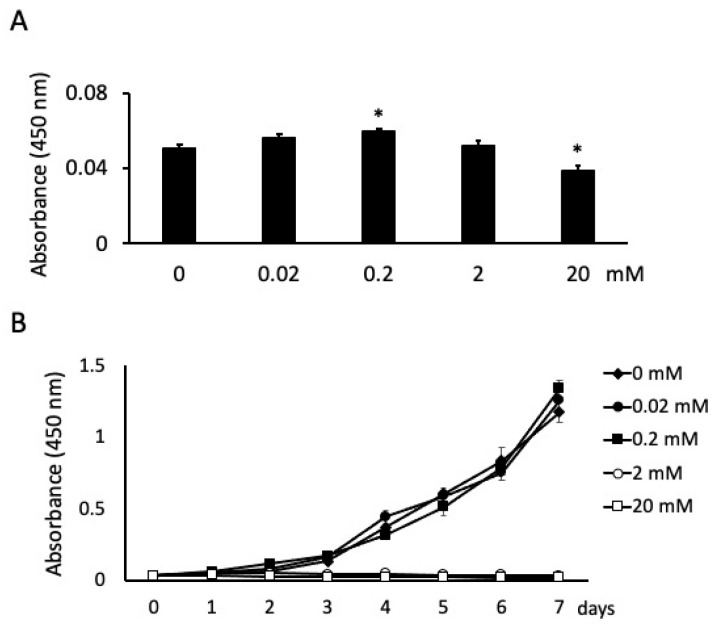
Effects of butyric acid (BA) on cell proliferation. Cells were incubated with several concentrations of sodium butyrate (NaB). Cell proliferations were measured at 24 h (**A**) and every 24 h until 7 days (**B**). Values are the mean ± standard deviation from eight independent experiments. * Significantly different from control cells (0 mM) (*p* < 0.05).

There were significant differences in cell proliferation between the cells stimulated with BA at different concentrations at 24 h. Specifically, the number of cells increased and decreased significantly at 0.2 mM and 20 mM NaB, respectively (Figure 1A). The absorbance of unstimulated cells was 0.0508; the absorbance of stimulated cells was 0.0563, 0.06, 0.052 and 0.039 for 0.02, 0.2, 2 and 20 mM NaB stimulation, respectively (Figure 1A). Cell proliferation was also measured for 7 days. At high NaB concentrations of 2 and 20 mM, cells exhibited cytotoxicity and did not grow from the second or first day, respectively. However, cell proliferation was unaffected at 0.02 mM NaB and significantly increased at 0.2 mM NaB after 7 days of incubation (Figure 1B).

### 3.2. Butyric Acid Upregulates Cell Migration in a Concentration-Dependent Manner

**Figure 2:**
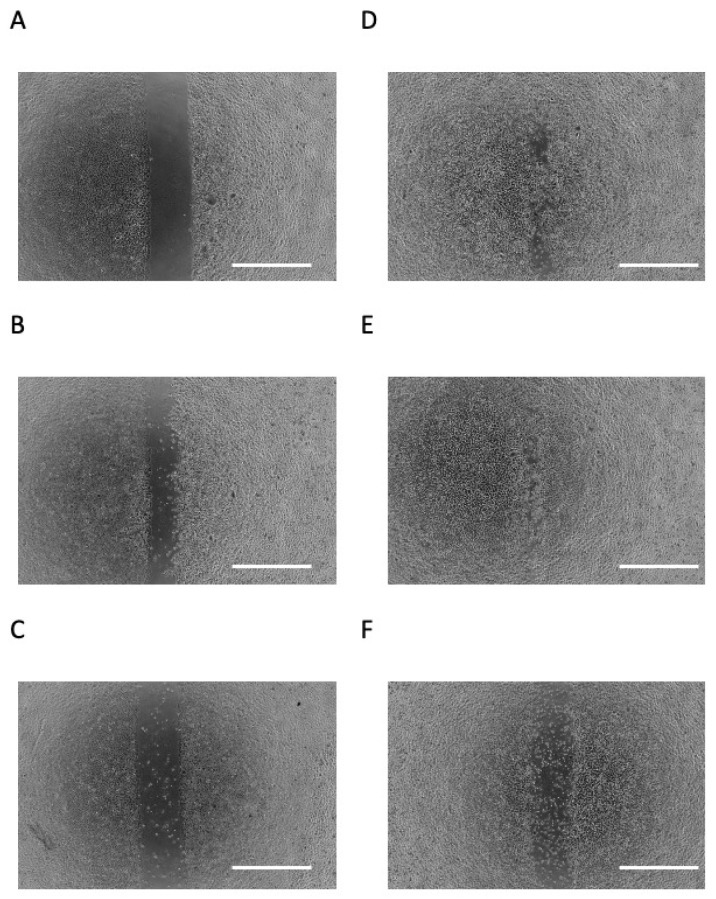
Effects of butyric acid (BA) on cell migration. Photomicrographs of scratch assay at day 0 (**A**), incubated with several concentrations of sodium butyrate (NaB), 0 mM (**B**), 0.02 mM (**C**), 0.2 mM (**D**), 2 mM (**E**) and 20 mM (**F**), for 1 day. All photomicrographs showed typical example of six independent experiments. Scale bar = 1 mm.

A scratch assay was performed as an in vitro mimic of epithelial down-growth (migration on hard tissue) during periodontal disease exacerbation. As shown in Figure 2A,B, cells migrated to the cell-free area after 24 h of culture. When the cells were incubated for 24 h with 0.2 mM or 2 mM NaB, they appeared to migrate to the cell-free area more so than unstimulated cells (Figure 2B,D,E). However, when cells were stimulated with 20 mM NaB, they did not appear to migrate to the cell-free area, and many dead and floating cells were observed (Figure 2F).

### 3.3. Butyric Acid Enhances Cell Attachment

**Figure 3:**
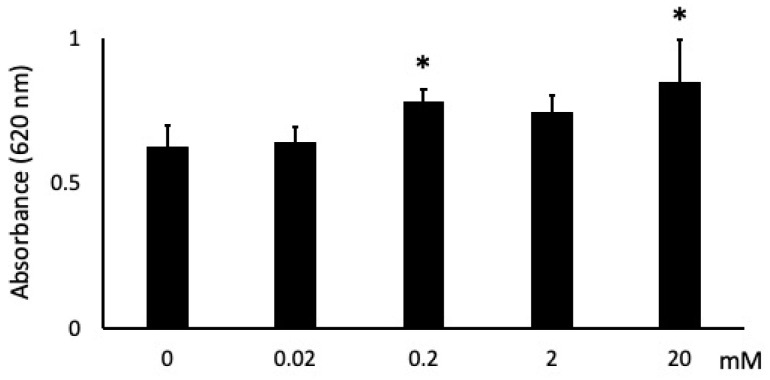
Effects of butyric acid (BA) on cell attachment. Cells were incubated with several concentrations of sodium butyrate (NaB), 0 mM, 0.02 mM, 0.2 mM, 2 mM and 20 mM, for 1 h. * Significantly different from control cells (0 mM) (*p* < 0.05). Values are the mean ± standard deviation from eight independent experiments.

Cells stimulated with 0.02 mM showed no change, 0.2 and 20 mM NaB showed ~25% and ~35% more cell attachment, respectively, than unstimulated cells at 1 h (Figure 3). In addition, while 2 mM NaB had no significant effect on attachment, it did result in an increasing attachment trend.

### 3.4. Effects of Butyric Acid on Adhesion Molecule Expression

**Figure 4:**
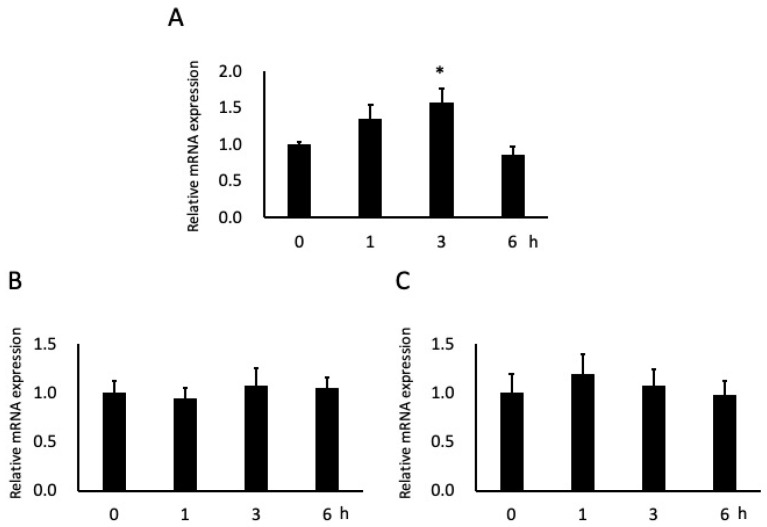
Effects of butyric acid (BA) on mRNA expressions of adhesion molecules. Cells were incubated with 0.2 mM sodium butyrate (NaB). Total RNAs were extracted from the beginning of the experiment (0 h), 1 h, 3 h and 6 h. Intercellular adhesion molecule-1 (ICAM-1) (**A**), integrin α6 (**B**) and integrin β4 (**C**) mRNA levels are shown relative to glyceraldehyde-3-phosphate dehydrogenase (GAPDH), the internal control. * Significantly different from control cells (0 mM) (*p* < 0.05). Values are the mean ± standard deviation from six independent experiments.

After 3 h of stimulation with 0.2 mM NaB, ICAM-1 mRNA expression in cells significantly increased (by ~60%); this expression also tended to increase after 1 h of stimulation but not to significant levels, and tended to be downregulated at 6 h (Figure 4A). Although the mRNA expression of integrin α6 and integrin β4 tended to increase after 3 h and 1 h of stimulation, respectively, the effects were not significant (Figure 4B, C).

### 3.5. CAGE and GO Term Enrichment Analysis

Given the significant increase in gene expression shown in Figure 4A, total RNA was extracted from these cells and CAGE and GO term enrichment analysis were performed. CAGE analysis revealed 75 upregulated and 96 downregulated genes in the cells after 3 h stimulation with 0.2 mM NaB. GO term enrichment analysis indicated that the genes upregulated >4-fold in the cells predominantly participated in cell migration and proliferation (Table 1).

## 4. Discussion

For the first time, the results of the present study show that BA participates in the progression of periodontitis by increasing the proliferation, migration and attachment of the junctional epithelium. By observing cell proliferation, we investigated the effects on tissue destruction involved in the progression of periodontal disease. In the cell proliferation and inhibition assay, low and high concentrations of NaB slightly increased cell proliferation and inhibited cell growth, respectively; thus, BA has opposing properties depending on concentration. This result supports previous studies in which intestinal cells [44], colonic epithelial cells [45], several oral cancer cells [46], and gingival epithelial cells [30] were used.

In the present study, we also observed the conflicting effects of BA on cell migration. Based on this experiment, we considered the effect on the down-growth of the junctional epithelium. Specifically, cell migration did not increase with the highest tested NaB concentration, 20 mM; indeed, many floating cells were observed, suggesting cell death. In contrast, cell migration increased with NaB stimulation at 0.2 and 2 mM. Interestingly, when cells were stimulated with 2 mM NaB, there was no significant change in cell proliferation after 24 h, but migration did increase. Therefore, it was suggested that low concentrations of BA may contribute to the down-growth of the junctional epithelium.

We speculated that cell attachment was involved in the observed cell migration. This is because strengthening adhesion may promote cell migration. In our investigation, cell adhesion tended to increase in a concentration-dependent manner. This may be related to changes in the expression of cell adhesion factors, three of which were also examined here by qPCR, ICAM-1, integrin α6 and integrin β4, having previously been reported in gingival epithelial cells [30]. qPCR can quantify the level of mRNA, which is not possible with conventional PCR, so it is possible to compare the expression levels of mRNA.

Our results generally support those of Takigawa et al. [30] but with some differences observed, i.e., both studies found a significant increase in ICAM-1 expression but we found no significant changes in integrin α6 and integrin β4 expression. However, increased ICAM-1 expression was observed earlier in junctional epithelial cells than the 6 h stimulation required in gingival epithelial cells; in contrast, expression tended to be downregulated at 6 h in junctional epithelial cells. Integrin α6 and integrin β4 are combined and function as a laminin receptor; they strongly promote cell adhesion to the extracellular membrane as well as migration and proliferation [39,47]. ICAM-1 is a factor involved in cell−cell adhesion and it plays a role in leukocyte migration. When inflammation occurs in periodontal disease, inflammatory cells such as leukocytes move to the gingival sulcus and cause an immune reaction [48]. Thus, our results suggest that the junctional epithelium reacts faster than the gingival epithelium, probably because it is closer to the periodontal tissue destruction site and dental plaque.

In GO term enrichment analysis of the genes and promoters for which expression was altered by NaB stimulation, the GO terms immune response, positive regulation of PI3K signaling, MAPK cascade, positive regulation of epithelial cell proliferation, cytokine activity, and growth factor activity were predominantly enriched. These GO terms are associated with cell proliferation and migration; thus, these findings support our cell proliferation and migration assay results. Since BA is a short-chain fatty acid, it has a low molecular weight and can easily enter cells. It also acts as a signaling molecule with biological effects, one of which is the epigenetic regulation of host gene expression by histone deacetylase (HDAC) inhibition [49]. We speculate that HDAC inhibitory action promoted the transcription of genes involved in cell proliferation and migration in the present study, leading to down-growth of the junctional epithelium. However, most of the genes associated with cell proliferation and migration were upregulated; the reason for this is unknown and will require further investigation. Another biological function of BA is the control of regulatory Tregs [26]; thus, additional studies are required to assess the possibility of BA-induced HDAC inhibition at the junctional epithelium and/or suppression of Tregs.

In summary, the effects of BA on the junctional epithelium reported here suggest that periodontopathic bacteria-produced BA may be involved in periodontal tissue destruction at high concentrations, while at low concentrations it potentially participates in the progression of periodontal disease through excessive increased proliferation, migration and attachment of the junctional epithelium.

## Figures and Tables

**Table 1:** A list of the gene ontology term enrichment analysis among over 4-fold upregulated genes in JE-1 stimulated with 0.2 mM NaB.

Category	Term	Count	Gene ID	*p*-Value
GOTERM_BP_DIRECT	Immune response	3	Ccl5, Il7, Tnfsf13	4.60 × 10^−2^
GOTERM_BP_DIRECT	Positive regulation of phosphatidylinostiol 3-kinase signaling	2	Ccl5, Tgfb2	7.80 × 10^−2^
GOTERM_BP_DIRECT	Neutrophil chemotaxis	2	Ccl5, Epgn	8.40 × 10^−2^
GOTERM_BP_DIRECT	MAPK cascade	2	Ccl5, Tgfb2	8.40 × 10^−2^
GOTERM_BP_DIRECT	Positive regulation of epitherial cell proliferation	2	Ccl5, Epgn	9.20 × 10^−2^
GOTERM_CC_DIRECT	Extracellular space	7	Actg1, Aga, Ccl5, Epgn, Il7, Tgfb2, Tnfsf13	9.90 × 10^−3^
GOTERM_MF_DIRECT	Cytokine activity	4	Ccl5, Il7, Tgfb2, Tnfsf13	2.10 × 10^−3^
GOTERM_MF_DIRECT	Growth factor activity	3	Epgn, Il7, Tgfb2	1.30 × 10^−2^
GOTERM_MF_DIRECT	Protein self-association	2	Aga, Ccl5	7.00 × 10^−2^

## Data Availability

Not applicable.
